# Plasmonics of Diffused Silver Nanoparticles in Silver/Nitride Optical Thin Films

**DOI:** 10.1038/s41598-019-56719-x

**Published:** 2019-12-27

**Authors:** Yufeng Ye, Joel Y. Y. Loh, Andrew Flood, Cong Y. Fang, Joshua Chang, Ruizhi Zhao, Peter Brodersen, Nazir P. Kherani

**Affiliations:** 1 Department of Electrical and Computing Engineering, University of Toronto, 10 King’s College Road, Toronto, ON M5S 3G4 Canada; 2 Division of Engineering Science, University of Toronto, 40 Saint George Street, Toronto, ON M5S 2E4 Canada; 3 Department of Chemical Engineering and Applied Chemistry, University of Toronto, 200 College Street, Toronto, ON M5S 3E5 Canada; 4 Ontario Centre for the Characterisation of Advanced Materials, University of Toronto, 200 College Street, Toronto, ON M5S 3E5 Canada; 5 Department of Materials Science and Engineering, University of Toronto, 140 College Street, Toronto, ON M5S 3E4 Canada; 6 Present Address: Research Laboratory of Electronics, Massachusetts Institute of Technology, 50 Vassar Street, Cambridge, MA 02142 USA

**Keywords:** Nanoparticles, Optical physics

## Abstract

Metal-dielectric multilayers are versatile optical devices that can be designed to combine the visible transmittance of dielectrics with the electronic properties of metals for plasmonic and meta-material applications. However, their performances are limited by an interfacial optical absorption often attributed entirely to the metal surface roughness. Here, we show that during deposition of AlN/Ag/AlN and SiN_x_/Ag/SiN_x_ multilayers, significant diffusion of Ag into the top dielectric layer form Ag nanoparticles which excite localized surface plasmon resonances that are primarily responsible for the interfacial optical absorption. Based on experimental depth profiles, we model the multilayer’s silver concentration profile as two complementary error functions: one for the diffused Ag nanoparticles and one for the interface roughness. We apply the Maxwell-Garnett and Bruggeman effective medium theories to determine that diffusion characteristics dominate the experimental absorption spectra. The newfound metal nanoparticle diffusion phenomenon effectively creates a hybrid structure characteristic of both metal-dielectric multilayer and metal-dielectric composite.

## Introduction

Metal-dielectrics are fundamental material platforms for photonic and electronic devices. Two important architectures include metal-dielectric multilayers, and metal-dielectric composites. Metal-dielectric multilayers are stacks of metal and dielectric layers simply fabricated by sequentially depositing the respective nanolayers. In addition to supporting the propagation of surface plasmons on the metal-dielectric interface^1^, metal-dielectric multilayers can be designed to utilize thin film interference (usually anti-reflection) to combine the high visible transmittance of dielectrics with the high electrical conductivity and infrared reflectance of metals; this leads to applications including flexible transparent conductors^2,3^, low-emissivity solar control coatings^4^, and optical filters^5,6^. However, the performance of such multilayer devices are often limited by an anomalous interfacial optical absorption^7,8^ which arises from non-ideal features that are not accounted for in standard device models. Many studies in the literature^7–10^ identify this interfacial absorption as the surface plasmon resonances (SPR) associated with the surface roughness of the metal film. But some other controlled-environment diffusion studies^11,12^ demonstrate that Ag, a common metal used in these multilayers, can be highly diffusive when the multilayers undergo post-deposition treatments such as annealing^11^ or UV photoillumination^12^.

However, few studies^13^ are devoted to the diffusion of Ag without post-deposition treatments, and to the best of our knowledge, there are three outstanding questions: firstly, whether there exists significant *in-situ* diffusion during deposition (i.e. without the post-deposition treatments^11,12^); secondly, what is the morphology of the diffused silver and its associated plasmonic mode; and thirdly, what is the appropriate optical model for the plasmonic effect of the diffusion phenomenon.

Herein, we report on the silver – hydrogenated aluminium nitride (AlN)^14,15^ and silver – silicon nitride (SiN_x_) systems in which we show significant silver diffusion and nanoparticle formation upon fabrication of the AlN/Ag/AlN and to SiN_x_/Ag/SiN_x_ multilayers. We observe the intense localized surface plasmon resonance (LSPR)^16^ peaks typically associated with metal-dielectric composites^17^ and find that they can only be accounted for by Ag nanoparticle diffusion. Thus, the multilayer is effectively a hybrid metal-dielectric multilayer-composite structure and can potentially serve as the basis for a novel synthesis method for nanostructures^18^ that exploit *in-situ* Ag diffusion and nanoparticle formation for applications such as optical switching^19,20^ and memristor devices^21,22^, or applications that combine^23^ SPR and LSPR such as ultra sensitive immunoassays^24^, DNA detection^25^, and enhanced surface plasmon-coupled emission^26^.

## Results

### Plasmonic absorption from Ag nanoparticles at AlN/Ag

**Figure 1:**
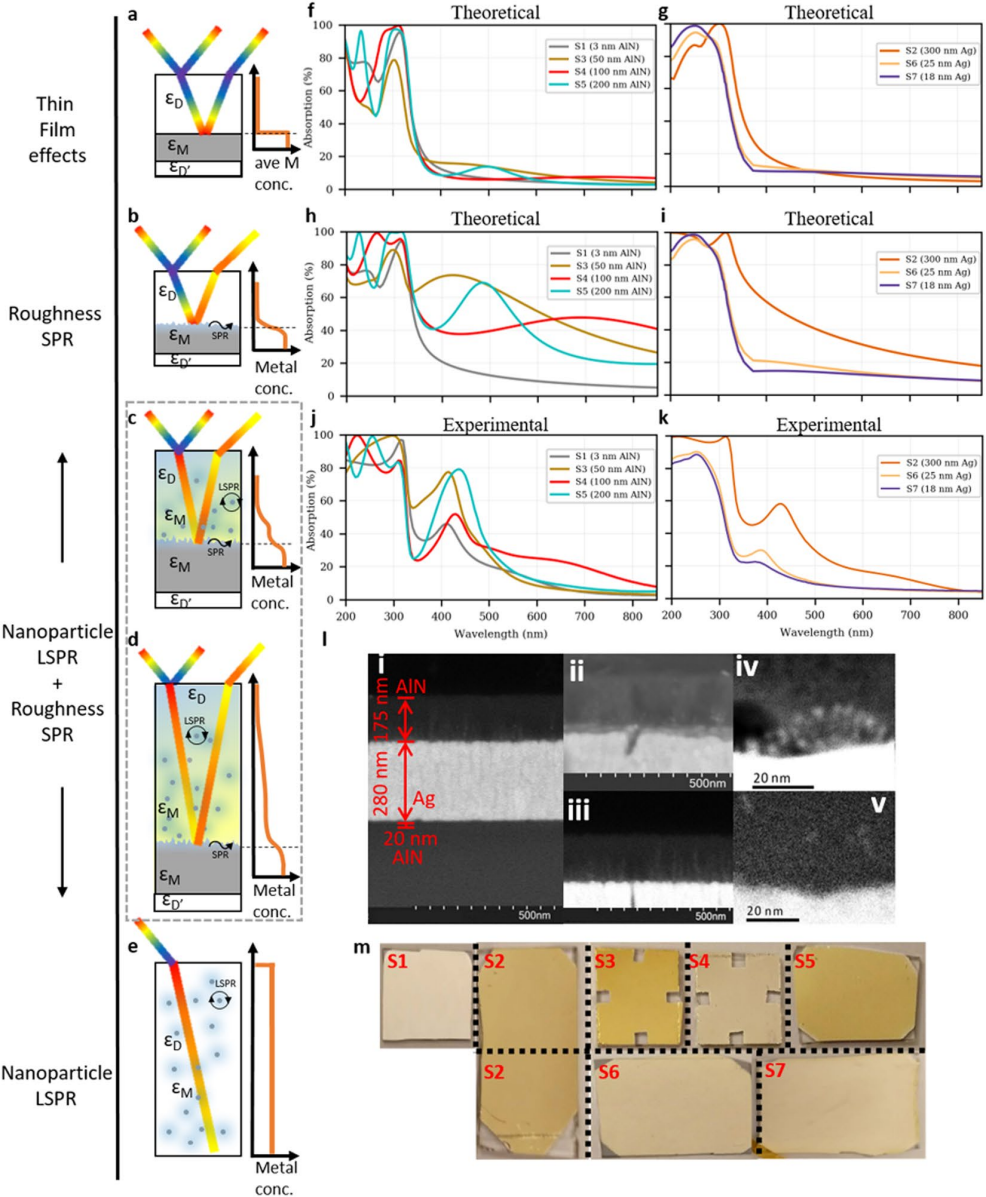
Optical role of diffused Ag nanoparticles in AlN. (**a–e**) Conceptual illustrations showing the various morphological regimes which cause optical absorption in metal-dielectric films, from **(a)** the simplest thin film interference effects in the ideal metal-dielectric multilayer to **(b)** the conventional SPR from surface roughness at the metal-dielectric interface, to **(e)** a form of metal-dielectric composite with LSPR from metal nanoparticles uniformly dispersed in a dielectric, and in this study, (**c,d**) LSPR of diffused metal nanoparticles with a concentration gradient within the dielectric layer. The average metal concentration profiles are sketched on the right to summarize the central difference between the scenarios. (**f–i**) Simulated absorption spectra of the fabricated AlN/Ag/AlN multilayer samples in Table 1: assuming **(f,g)** ideal sharp interface; and **(h,i)** with additional surface roughness; **(f, h)** varying thickness of the top AlN layer on 300 nm thick Ag; and **(g,i)** varying Ag layer thickness sandwiched between 20 nm AlN layers. (**j,k**) Experimentally measured absorption spectra of samples. **(l)** (i–iii) SEM BSE cross sectional images showing high intensity specks associated with the Ag nanoparticles within the AlN; (iv,v) STEM dark field images show smaller silver nanoparticles (iv) 10–20 nm away and (v) ~50 nm away from the interface. (**m**) Photographs showing the colors of the samples.

We begin by first focusing on AlN as the dielectric that forms an interface with Ag, then in a later section we generalize these results by demonstrating their applicability to SiN_x_. Table 1 details the AlN/Ag/AlN multilayered samples which were fabricated with controlled variations in thicknesses of the top AlN layer and the Ag layer. The intense golden coloration of the films (Fig. 1m) was immediately visible following sputter deposition of the samples and persisted for at least 14 months. UV-Vis absorption spectra of these samples (Fig. 1j,k) reveal the strong absorption peaks in the violet wavelengths (380–450 nm) which give rise to the observed complementary golden color^7^.

**Table 1:** All fabricated thickness combinations of AlN/Ag/AlN thin film stacks on glass and silicon substrates.

Label	Top AlN layer [nm]	Ag layer [nm]	Bottom AlN layer [nm]
S1	3	300	20
S2	20	300	20
S3	50	300	20
S4	100	300	20
S5	200	300	20
S6	20	25	20
S7	20	18	20

Supplementary Materials.

Supplementary materials are provided in a separate file. Figures S1−S7. Tables S1–S6.

Samples S1, S2, S3, S4, S5 have controlled variations in top AlN layer thicknesses for fixed bottom 300 nm Ag/20 nm AlN layers, while samples S2, S6, S7 have controlled variations in middle Ag layer thicknesses between fixed 20 nm AlN layers.

When Ag nanoparticle diffusion and its associated LSPR phenomenon are not accounted for, standard thin film interference models (Fig. 1a) predict much weaker, broader, and more red-shifted absorption peaks (Fig. 1f,g) than those observed (Fig. 1j,k). However, the predicted interference maxima and minima (that alternate with increasing dielectric thickness) correctly dominate the ranking of absorption intensities among samples S1–S5 at around 500 nm (Fig. 1j). For detailed discussion, see Supplementary Information – Absorption Spectra.

Given that interference effects inadequately account for the observed absorption spectra, surface roughness effects need to be included in the model (Fig. 1b). Following standard practice, we introduce a roughness layer with an estimated thickness of 15 nm, as determined from the cross-sectional TEM images in Fig. 1l (Supplementary Information – Roughness Estimation). The effective index of the roughness layer is computed from the Bruggeman (BG) Effective Medium Theory (EMT) assuming a conventional^27,28^ 50:50 split between Ag and AlN material contribution by volume. The corresponding scattering matrix calculated results in Fig. 1h,i are closer to the observed spectra in Fig. 1j,k. We see that the additional absorption in the visible due to the SPR associated with the rough interface is of the right order of magnitude, but the roughness model cannot produce the observed sharp peaks and tends to overestimate the absorption in the infrared. See Supplementary Information – Scattering matrix method for relevant details.

To better understand the underlying cause of the absorption, cross-sectional Scanning Electron Microscopy (SEM) images and Scanning Transmission Electron Microscopy (STEM) of sample S5 were taken in Back-Scattered Electron (BSE) mode (Fig. 1l). These images show the presence of Ag nanoparticles of ~5–10 nm radii deep into the AlN layer, which appear as high intensity specks owing to the atomic number contrast between the two materials. The LSPR excited by these Ag nanoparticles would significantly enhance the absorption properties of our samples. The resulting absorption is characteristic of both the SPR-dominated planar metal-dielectric interface (Fig. 1a,b) and the LSPR-dominated dielectric-embedded metal nanoparticles (Fig. 1e), as shown in Fig. 1c,d. The resulting hybrid structure is anticipated to have optical properties that depend on the concentration profile of Ag in the structure, as illustrated conceptually in Fig. 1c,d.

### Complementary Error Function (*erfc*) ToF-SIMS depth profiles

**Figure 2:**
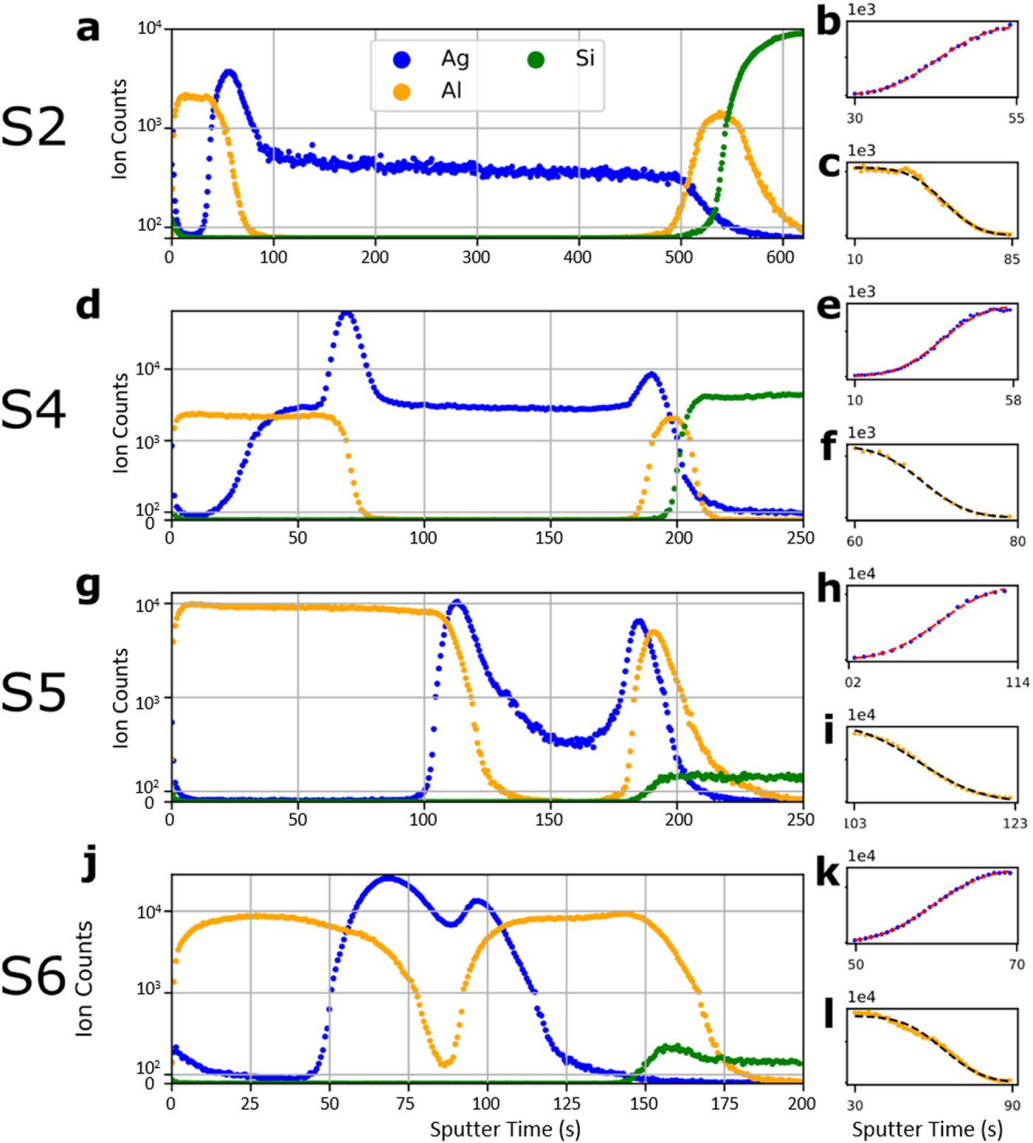
ToF-SIMS profile with analysis for samples S2–S6. (**a,d,g,j)** The depth profiles of the relevant ion species. To minimize the visual impact of Ag peaks due to interface artefacts, the ion counts above 1000 are in log scale. (**b,c,e,f,h,i,k,l)** The regression fits to the **(b,e,h,k)** Ag ion counts and **(c,f,i,l)** to the Al ion counts, with an *erfc* model.

To quantify the spatial distribution of Ag nanoparticles, Time of Flight - Secondary Ion Mass Spectroscopy (ToF-SIMS) depth profiles were taken for samples S2, S4, S5, and S6, which are shown in Fig. 2a,d,g,j, respectively. In Fig. 2, the positive secondary ion counts corresponding to the constituent elements of the film (i.e., Ag, Al, Si in AlN/Ag/AlN/Si) are plotted as a function of the ToF-SIMS sputter time, which serves as a proxy for the distance from the surface of the film. In Fig. 2a,d,g,j, we identify a region of steady ion counts as the layer of the material associated with the ion type^29^. The presence of extremely high Ag ion count, peaking where the Al ion profile undergoes drastic change, is an interfacial artefact^30–32^ likely due to the steep change in oxidation states at the interface and as such does not represent a peak in the concentration of silver. Similarly, the rise in Ag ion counts near the surface of the film is likely due to local changes in the oxidation linked to oxygen contamination of the open surface. For more in-depth discussions on data interpretation, see Supplementary Information - ToF-SIMS.

We model and fit the gradients of ion counts to the complementary error function, *erfc(z)*, which is both a well-known solution to Fick’s Second Law of Diffusion^33^ and a commonly observed concentration profile at interfaces^13,32^. The resulting fits, shown in Fig. 2b,e,h,k and Fig. 2c,f,i,l for Ag and Al ions, respectively, match the *erfc* model closely (see Supplementary Information - ToF-SIMS for statistics). Because the respective concentrations of Ag and AlN sum up to unity, we can convert a decreasing AlN *erfc* transition to an increasing Ag *erfc* transition. By combining this converted Ag *erfc* transition with the plotted Ag *erfc* transition, we find a two-step *erfc* profile as conceptually illustrated in Fig. 1c,d. Given the observed Ag nanoparticle diffusion (Fig. 1l), and the natural description of diffusion concentration profiles as *erfc* functions, we attribute one of the two *erfc* functions to the concentration profile of diffused Ag nanoparticles and the other *erfc* function to the interfacial transition (including roughness) between the AlN and Ag. Then, the percentage volume of Ag, *f(x)*, at any depth *x* is given by a linear combination of the two *erfc* functions:

1 $$f(x)={A}_{1}erfc[{S}_{1}(x-{C}_{1})]+{A}_{2}erfc[{S}_{2}(x-{C}_{2})].$$

Here A_1_ and A_2_ are any pair of positive amplitudes that yield a physically realistic *f(x)* that approaches 0% and 100% at the two *x* limits. The *S* parameters control the steepness of the *erfc* function and the *C* parameters control the centres of the *erfc* function; together, *S* and *C* provide the linear transform *S* (*x* − C) for the sputter time *x*, thereby enabling *erfc*(*x*)′s of different widths and centres. This depth profile model is used in the upcoming optical modelling section.

### XPS depth profile

**Figure 3:**
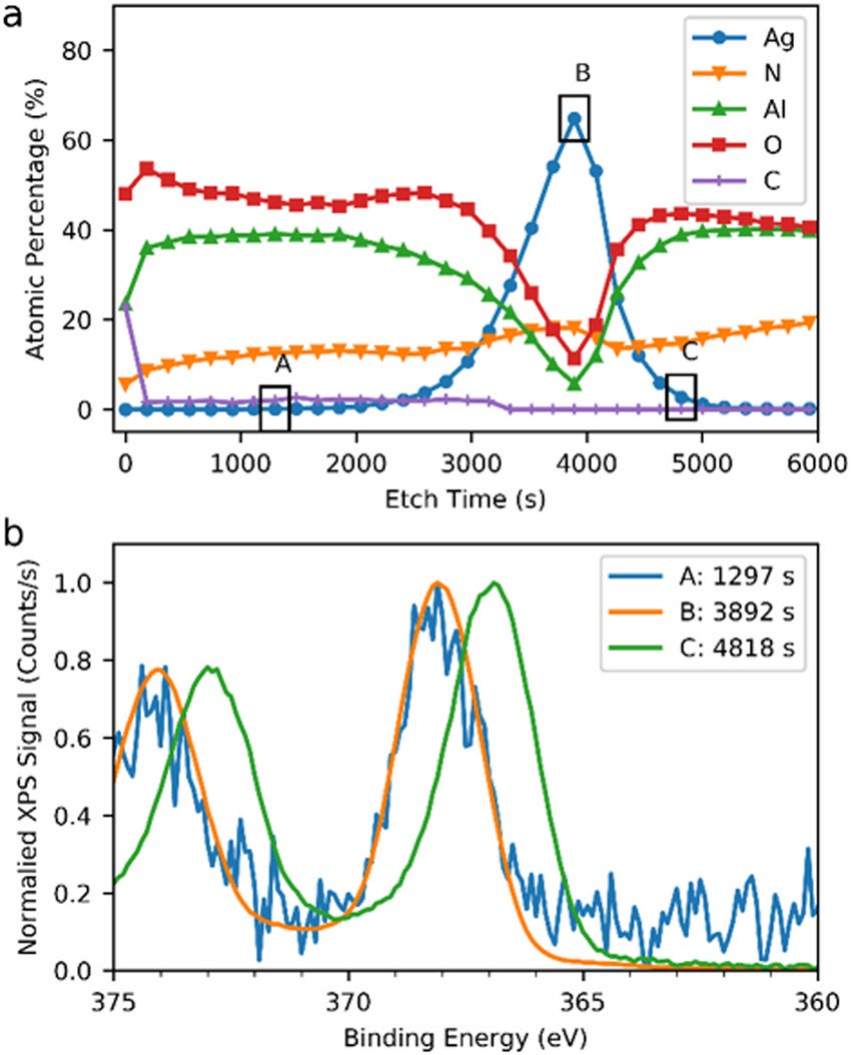
XPS Data for Sample S6. (**a)** XPS depth profile of atomic concentration **(b)** XPS binding energies for the silver 3d3/2 and 3d5/2 peaks at selected depths. 1297s is within the top AlN layer, 3892s is within the silver film, and 4818s is within the seed AlN layer, close to the silver interface. The analytical probe includes contribution of signals from the top 8–10 nm of the bottom of the sputter crater. Convoluted with this are sputtering effects.

To gain further insight into the nanoparticle formation mechanism, an XPS depth profile of sample S6 was taken (Fig. 3a). Based on the relative atomic concentrations, we calculated the Ag volume fraction as ~0.24% at an etch time of 1297s (see Supplementary Information – Calculation of Silver Volume Concentration for details). In addition, the binding energies of the silver 3d^5/2^ and 3d^3/2^ electrons provide some insight into the chemical state of silver in this structure. As can be seen in Fig. 3b, close to the interface of the bottom AlN and silver layers, the 3d^5/2^ and 3d^3/2^ peaks are at 367.0eV and 373.0eV, respectively. This is consistent with AgO^34,35^. Ag_2_O peaks would have slightly higher energies^34,35^. Throughout the silver film and into the AlN top layer, the 3d^5/2^ and 3d^3/2^ peaks are closer to 368.1eV and 374.1eV. This is more in keeping with bulk silver binding energies and therefore silver-silver bonding^34,35^. These binding energies are therefore further evidence of the silver diffusion being in the form of nanoparticles or clusters.

### Calculation of optical absorption

We now construct an optical model to account for both the diffused Ag nanoparticles and the associated LSPR as well as the rough interface and the associated SPR, for samples S3–S6 (see also Samples S1 and S2 in Supplementary Information). Based on the analysis of ToF-SIMS profiles, we discretize the Ag concentration profile, *f(x)* — as formulated in Eq. (1), into 300 layers with increments of 0.333 vol.% Ag per discrete step. The vol.% of Ag is assumed to be constant over each discrete thickness step. We can then compute the absorption spectra of the entire multilayer using the EMT models of Maxwell-Garnett^36,37^ (MG) and Bruggeman (BG). With the MG model, the effective dielectric constant <ε> can be approximated as:

2 $$\langle \varepsilon \rangle ={\varepsilon }_{D}+\frac{f{\varepsilon }_{D}({\varepsilon }_{M}-{\varepsilon }_{D})}{{\varepsilon }_{D}+(1-f)({\varepsilon }_{M}-{\varepsilon }_{D})L}{\varepsilon }_{D}$$

The depolarization coefficient *L* accounts for the non-ideal eccentricity of the spherical inclusions. The MG model is used extensively in the modelling of metal-dielectric composites because of its success in predicting LSPR absorption^38^. In contrast, the BG model applies for large and arbitrarily shaped inclusions of fractional content *f* such that *f* is large compared to the values for the MG model. The resulting effective dielectric constant $\langle \varepsilon \rangle$ is the solution of^39^:

3 $$(1-f)\frac{{\varepsilon }_{D}-\langle \varepsilon \rangle }{{\varepsilon }_{D}+2\langle \varepsilon \rangle }+(f)\frac{{\varepsilon }_{M}-\langle \varepsilon \rangle }{{\varepsilon }_{M}+2\langle \varepsilon \rangle }$$

The BG model does not predict LSPR effects which are localized near the surfaces of spherical particles^38,40^.

**Figure 4:**
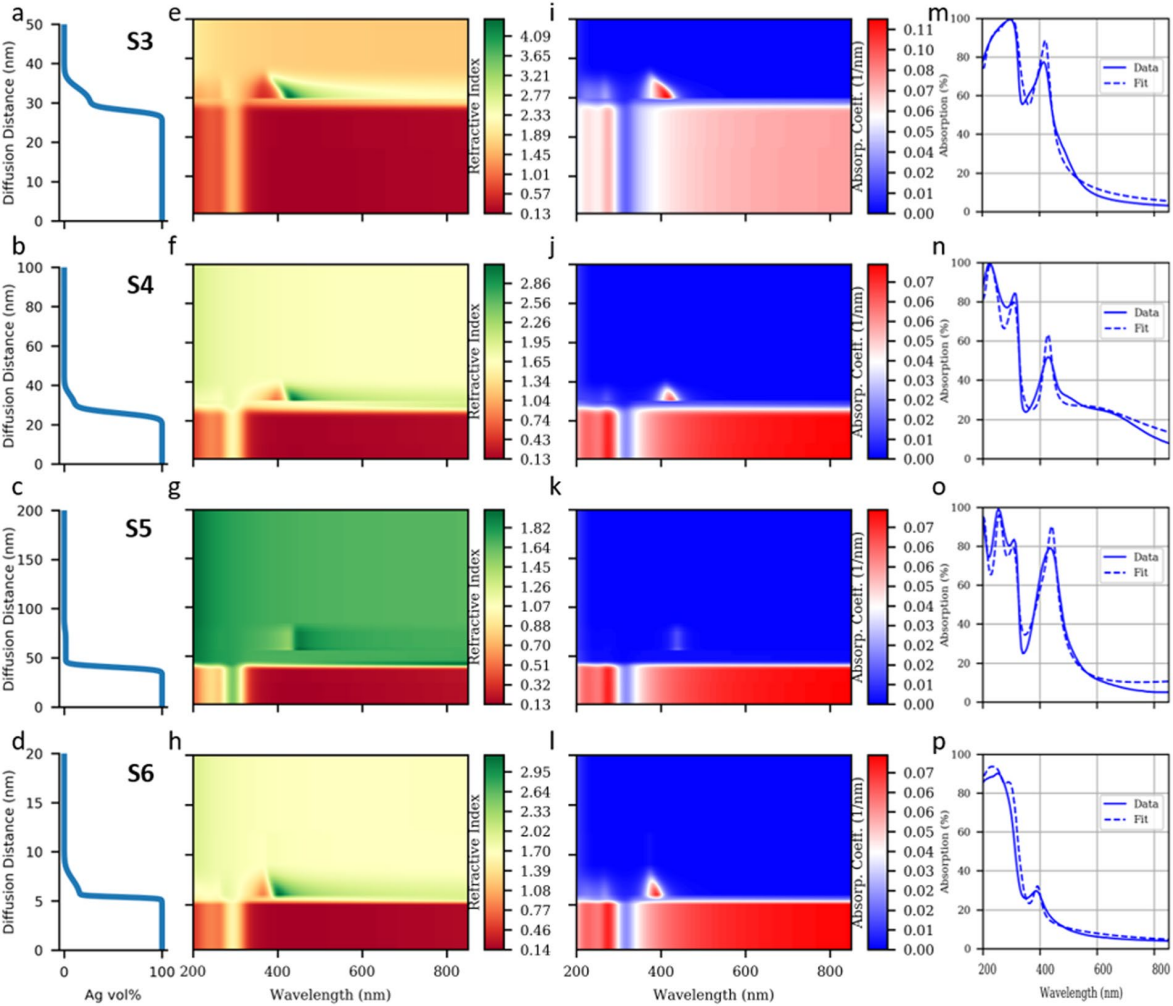
Concentration profile regression fit results for samples S3–S6 (rows 1 to 4, respectively). (**a**–**d)** Best fit concentration profiles of Ag in the top AlN layer. The diffusion distance is the upward distance from the expected Ag layer surface. **(e–h)** Refractive index spectra and (**i–l)** absorption coefficient spectra obtained by BG and MG EMT in the top AlN layer, corresponding to the concentration profile. LSPR signatures are found in regions with both sharp variation in the refractive index spectrum and high absorption coefficients. (**m–p)** UV-Vis absorption spectra: experimental and that calculated by scattering matrix using refractive indices from EMT. Note that the diffusion distance axes have different scales and that the refractive index and absorption coefficient spectra have different color scales across the samples due to the large variations among them.

Thus, we appropriately apply the BG model to the *erfc* function representing regimes dominated by roughness induced SPR and the MG model to the *erfc* function representing regimes dominated by diffused nanoparticle induced LSPR. Figure 4 shows the results of the regression fit in which *f(x)* is varied to match the calculated absorption spectra with the experimental absorption spectra. For more details on the regression algorithm, see Supplementary Information – Absorption Fit from Diffusion.

The best-fit Ag concentration profiles (Fig. 4a–d) yield excellent matches with the experimental data shown in Fig. 4m–p. In Fig. 4a–c, we see that the concentration profile of diffused Ag nanoparticles, represented by the smaller *erfc* function, spreads out further from the Ag surface and assumes lower percentages in thicker top dielectric layer samples. Because thicker dielectric layers require longer sputter deposition time, there is more time for Ag close to the interface to diffuse deeper into the dielectric, as governed by Fick’s Second Law^33^. Further inspection of Fig. 4a–c reveals that the effective interface between Ag and AlN, defined as the centre of the first *erfc* function from the bulk silver layer, shifts further into the dielectric layer. This is a well known phenomenon associated with the interdiffusion of two species with very different diffusion coefficients^33^. As a result, the effective thickness of the AlN layer shrinks, which causes the UV interference peaks to shift from the predicted profiles (i.e., for ideally sharp interfaces), evident from Fig. 1f,j.

Figure 4e-h,i–l show the calculated spectral refractive index and absorption coefficient profiles. The bottom of the contour plots (with silver concentration approaching 100%) corresponds to the bulk silver region, which is strongly reflective. Next, in the roughness region where the silver concentration drops significantly, it is expected that no LSPR peaks are produced because of the choice of applying the BG EMT model. Instead, the LSPR peaks are produced by the Ag in the diffusion region and are modelled by the MG EMT (see Supplementary Information – Roughness LSPR for more discussion). The LSPR peaks manifest in the violet wavelengths as sharp spectral variations in the refractive index spectrum and sharp peaks in the absorption coefficient spectrum. These LSPR peaks have intensity and width that depend strongly on the shapes of their corresponding *erfc* function concentration profile. For lower metal concentration, the MG EMT model predicts a more blue shifted and lower intensity LSPR peak^41^. Hence, as the diffusion extent increases (distance on the vertical axes of Fig. 4a–d) and the silver concentration decreases to zero, the associated band of high absorption coefficients in Fig. 4i–l blue shifts and narrows, forming an “absorption triangle” region in the distance and wavelength plane. Contrasting the two right-most columns of Fig. 4, we see that the “absorption triangle” (Fig. 4i–l) matches the shape of the experimental absorption peak (Fig. 4m–p).

For sample S5, Fig. 4c predicts that the silver diffusion profile is extended quite a bit (~30 nm) into AlN but shallow (of the order of 1%). Because of the small (~1% to 0%) variation in the diffused silver concentration in the top layer, there is less blue shifting and variation in the width of the “absorption triangle” in Fig. 4k, which translates to a relatively symmetric experimental absorption peak in Fig. 4l. Despite the lowered absorption coefficients in Fig. 4k due to low silver concentrations, the vast distance of silver diffusion dominates and leads to a very high experimental absorption peak intensity in Fig. 4o. For sample S6, the only sample in Fig. 4 with a thin Ag layer of 25 nm, we see that similar features to that of the other thick silver samples are exhibited, including the presence of an “absorption triangle”.

However, owing to the assumptions inherent to MG and BG EMTs^39^, some second order effects may be omitted - such as additional enhanced coupling that results from adjacent nanoparticles as well as between the nanoparticles and the roughness features of the metal surface. The lack of a fair treatment of the latter is a well-known problem^42^ from the discontinuity between the MG and BG EMT formalisms^38^, and manifests as the “sheath” or the sudden transition between the SPR and LSPR dominant regions in the contour plots of Fig. 4. We also note that the model should be further specialized to include additional parameters that account for the varied silver layer thickness due to diffusion and the interdiffusion at the second Ag/AlN interface. For more discussions related to Fig. 4, see Supplementary Information - Absorption Fit from Diffusion.

### Ag nanoparticle formation in SiN_x_/Ag

**Figure 5:**
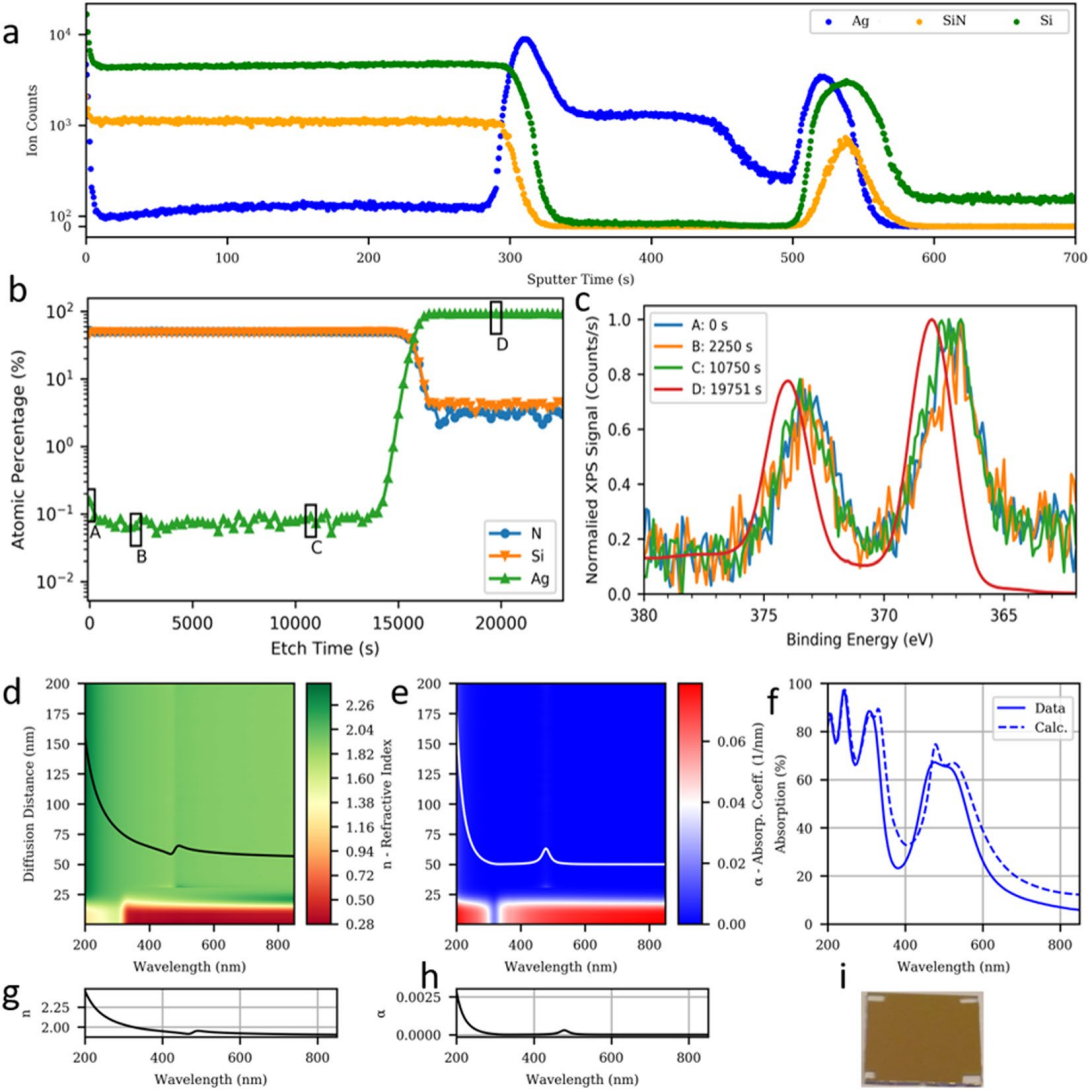
Ag nanoparticle formation in SiN_x_. (**a)** ToF-SIMS profile showing diffusion of Ag in the top SiN_x_ layer. Ion counts above 1000 are in log scale. (**b)** XPS depth profiles of Si, N and Ag. **c** XPS Binding Energy spectra of Ag at selected points within the stack at the surface (A), sub-surface (B), near interface (C), and in the Ag layer (D). There is a concentration of Ag within the SiN_x_ layer. (**d)** Refractive index spectra, (**e**) absorption coefficient spectra, and (**f**) computed absorption spectra as obtained by MG and BG EMT in the top SiN_x_ layer, corresponding to the concentration profile obtained from XPS. (**g)** Refractive index and h absorption coefficient spectrum computed from MG EMT with the representative 0.1 vol% diffused Ag nanoparticle in top SiN_x_. LSPR occurs at ~480 nm with the anomalous index dispersion and absorption peak. Scaled versions of (**g,h)** are also highlighted in (**d,e)** as black and white lines, respectively. (**i)** Image of the SiN_x_/Ag/ SiN_x_ stack on glass substrate.

We verify that the Ag nanoparticle formation in AlN/Ag/AlN is generalizable to other dielectrics by studying the SiN_x_/Ag/SiN_x_ multilayer. Figure 5 shows that with ToF-SIMs (Fig. 5a) and XPS (Fig. 5b) depth profile measurements, ~0.05% a.t. (~0.1% vol.) of Ag is present within the top SiN_x_ layer of a 200 nm SiN_x_/300 nm Ag/20 nm SiN_x_ sputter-deposited layers (Fig. 5i). The Ag concentration profile does not follow a conventional *erfc* function profile but has a steady concentration for ~135 nm of the top SiN_x_ thickness, which resembles the concentration profile of a limited source diffusion after a long period of time evolution^43^. As shown in Fig. 5c, the Ag 3d^5/2^ and 3d^3/2^ peaks in the silver film are close to 374.1 and 368.0 eV, which is similar to those observed in the Ag layer with AlN. At the surface and throughout the top nitride layer, the silver signal is noisier than in the AlN samples. However, it appears that there are additional lower energy Ag peaks (373.2 eV and 367.1 eV), along with the “bulk” silver peaks. These indicate significant oxidation of silver^34,35^, and are possibly due to the outer shell of the Ag nanoparticle oxidizing^44,45^.

Using the volumetric profile determined by the XPS depth profile measurement and SiN_x_ refractive index obtained from SE (see Supplementary information- SE of SiN_x_), we can cross validate the regression method in Fig. 4 by applying the same MG and BG EMT to directly calculate the refractive index and absorption coefficient variation over the thickness of the top SiN_x_ as shown in Fig. 5 d,e. An absorption peak and refractive index dispersion due to the diffused silver nanoparticle LSPR is seen at 475 nm wavelength. By applying MG only to the ~0.1% vol. diffused Ag profile, and BG to the SiN_x_/Ag interface, we computed a double peak absorption from 375 nm to 600 nm which closely matches the experimentally determined absorption profile of Fig. 5f. The higher wavelength component of the double peak, centered at 525 nm, is due to the roughness of the Ag/SiN_x_ interface (and its associated SPR) while the lower wavelength component, centered at 480 nm is due to the lower Ag concentration within the SiN_x_ layer (and its associated LSPR). The broad absorption peak gives rise to a brown color in the SiN_x_/Ag/SiN_x_ stack as shown in Fig. 5i.

## Discussion

With the existence of Ag nanoparticles confirmed via several types of characterization, we can contemplate its formation mechanism. We postulate that Ag atoms thermally diffuse from the bulk Ag film surface during deposition of the top nitride layer. This is supported by the *erfc* function depth profile as well as the common knowledge that Ag is a metal with a large diffusion coefficient^46^ and hence diffusion can occur during the relatively low temperature sputter deposition process. With regard to the mechanism of Ag nanoparticle formation, we hypothesize that our choice of nitride dielectrics, AlN and SiN_x_, suppresses the oxidation of silver which allows nanoparticle nucleation without the additional reduction that is usually required^47^. Here, the nitride matrix is superior to common oxide dielectrics because the formation of Ag_3_N is extremely unlikely (ΔG_f_ =314.4 kJ/mol)^48^. Also, the high oxygen affinity of Al and Si result in preferential formation of aluminum oxide and silicon oxide which effectively protect the diffusing silver from oxidation. Indeed, this is what we observe in the XPS measurements; we see that silver oxidation is insignificant in comparison to the significant aluminum oxide found in the multilayer structure.

In summary, we demonstrate *in-situ* silver nanoparticle formation due to diffusion at the sputter-deposited Ag/dielectric (AlN and SiN_x_) interfaces. The silver nanoparticles captured in SEM, STEM images have an *erfc* function spatial distribution derived from ToF-SIMS and XPS depth profiles. This creates a unique nanostructure wherein both SPR and LSPR modes coexist; this is especially apparent in the case of SiN_x_/Ag where the distinct peak wavelengths of the two plasmon modes are clearly visible in the spectra. Furthermore, we develop a physically realistic MG/BG EMT model that successfully accounts for the entire optical spectra of this nanostructure. Our findings provide both fundamental insights and a novel *in-situ* silver nanoparticle synthesis method that seamlessly integrates with conventional fabrication of planar metal-dielectric interfaces – which has enormous potential for the facile fabrication of devices with embedded nanoparticles for applications including light trapping^49^, biosensing^50^, transparent heater^51^, infrared detector^52^, and SPP-LSP coupling^24–26^.

## Materials and Methods

### Sputter deposition

Hydrogenated AlN and Ag thin films of varying thickness (as summarized in Table 1), on substrates of alkaline earth boro-aluminosilicate (Corning Eagle XG) 1.0-mm thick glass and (100) silicon wafer, were sputter deposited in an RF magnetron sputtering system using 99.99% pure Al and Ag targets, with 99.99% N_2_ and Argon, and 99.9% pure H_2_ (see Supplementary Information for details on substrate cleaning, deposition, and target pre-sputtering parameters). The sputtering chamber was cryogenically pumped to a base pressure of less than 1.8 E-6 Torr prior to any sputtering. Ag layers were deposited using only the Ag target with Ar and N_2_ gas flow ratio 8:8.6 sccm; AlN thin layers were deposited with reactive sputtering using the Al target with Ar, N_2_ and H_2_ gas flow ratio of 15:3.6:0.7 sccm. Supplementary Information Table S2 summarizes the deposition parameters of Ag and AlN thin films. The nominal thickness values of Ag films were obtained from a calibrated quartz crystal microbalance sensor inside the sputtering system. The nominal thickness and rate of deposition of AlN were determined using spectroscopic ellipsometry (SE) on single layer AlN films deposited on (100) silicon. Further, the film thicknesses were confirmed through cross-sectional SEM. The average deposition rates were then used to fabricate the various dielectric/metal stacks. For SiN_x_/Ag/SiN_x_ stacks, we reactively sputtered Si at 200W with N_2_ at a flow ratio of 50: 50 sccm Argon: N_2_ with a quartz crystal sensor determined 20 nm as the seed layer, sputtered Ag with N_2_ at a flow ratio of 8: 9 sccm Argon: N_2_ to 300 nm thickness, and reactively sputtered Si with N_2_ at 200W to a thickness of 200nm.

### Optical measurements and images

Spectroscopic ellipsometry (SE) measurements were taken at an angle of 60 degrees for wavelengths between 200 nm to 850 nm using a SOPRA GES-5E. SE regression fit was performed with the commercial software WinElli II and the results were used to estimate the nominal film thicknesses (see Supplementary Information – SE Results for details). A Perkin Elmer Lambda 1050 UV-Vis-NIR Spectrophotometer was used to carry out the transmittance and reflectance measurements at intervals of 5 nm (see Supplementary Information – UV-Vis for details). SEM images were obtained after etching the sample cross-section with table top precision ion milling (Hitachi Ion milling system IM4000). The STEM dark field images were obtained with Hitachi HF-3300 cold field-emission. Samples were prepared via Focused Ion Beam (FIB) by first depositing a tungsten protective layer. A 40 keV Ga ion beam with sequentially finer apertures was used to mill and thin the sample. After milling, but before thinning, a micro-sampling system was used to remove 3 samples of ~15 µm/5 µm/3 µm (length/height/width).

### ToF-SIMS and XPS depth profile

ToF-SIMS depth profiles, for positive secondary ions, were obtained on ION-TOF GmbH. (Muesnter, Germany) with low energy 500 eV O_2_ sputter beam and 30,000 eV Bi^+^ analysis beam for sample S2, S5 and SiN_x_/Ag/SiN_x_; and 1 keV O_2_ sputter beam and 60,000 eV Bi^3++^ analysis beam for sample S4. The crater size was 200 by 200 micron and secondary ion sampling area was 50 by 50 micron. EscaLab 250Xi XPS (Thermo Scientific, East Grinstead UK) with monochromatic AlKα X-rays (1486.68 eV) was used for the XPS analyses. The depth profile was obtained by etching the sample with Ar^+^ ion gun at 500 eV. The Avantage software program is used to post-process the data for quantification and spectroscopic interpretation.

### Scattering matrix and EMT modelling

Custom Python code was used to perform scattering matrix thin film multilayer calculations with BG and MG EMTs. Regression fit was performed with the Python scipy.optimize.curve_fit package.

## Supplementary information

Supplementary information.

## Data Availability

All the data presented and custom Python code used in this study are available from the corresponding author upon reasonable request.
