# Supplementary material for: Plasmonics of Diffused Silver Nanoparticles in Silver/Nitride Optical Thin Films
Source: Sci Rep. 2019 Dec 27;9:20227. doi: 10.1038/s41598-019-56719-x (PMC6934463; doi:10.1038/s41598-019-56719-x)
Supplement: Supplementary file 1 — Supplementary information. [file 41598_2019_56719_MOESM1_ESM.docx]

**Supplementary Materials to “Plasmonics of Diffused Silver Nanoparticles in Silver/Nitride Optical Thin Films”.**

**Cleaning of Substrates**

Substrates were ultrasonically cleaned with acetone, then isopropanol, and followed by deionized water for 10 minutes and were then thoroughly rinsed with deionized water and blown dry with nitrogen. The substrates were used immediately after cleaning.

**Sputter Deposition of Thin Films**

We used 3.00’’ diameter x 0.250’’ thick 99.99% pure Al, Ag, and Si targets, N_2_ Grade 4.8, Ar Grade 5.0 and H_2_ Grade 5.0 for both pre-sputtering and the actual deposition of thin films onto substrates.

**Pre-sputtering**

Pre-sputtering was carried out to clean the targets as shown in Table S1. The silver target was first pre-sputtered using pure Ar for about 20 nm (~60 s) or until the DC bias stabilized to remove possible contaminants on the target. The silver target was then pre-sputtered using a mixture of Ar and N_2_ (the same condition as our actual deposition of Ag shown in Table S2) for 20 nm (~60 s). The thickness of the silver was monitored by the calibrated QCM. The aluminum target was first pre-sputtered using pure Ar for at least 900s or until the DC bias stabilized to remove possible contaminants. Then the Al target was pre-sputtered using a mixture of Ar, N_2_, and H_2_ (the same condition as the actual deposition of AlN shown in Table S2) to ensure the top of the target was coated with the desired ceramic material.

**Table S1-a.** Pre-sputtering specifics for hydrogenated AlN

| Step | Target | Gas Ratio | | | Pressure (mTorr) | | RF Power (W) | | Nominal Time (s) |
| --- | --- | --- | --- | --- | --- | --- | --- | --- | --- |
|  |  | Ar (sccm) | N_2_ (sccm) | H_2_ (sccm) | Ignition | Process | Ignition | Process |  |
|  | Ag | 20 | 0 | 0 | 20.1 | 5.1 | 300 | 300 | 60 |
|  | Ag | 8 | 8.6 | 0 | 20.1 | 5.1 | 300 | 300 | 60 |
|  | Al | 20 | 0 | 0 | 20.1 | 5.1 | 250 | 250 | 900 |
|  | Al | 15 | 3.6 | 0.7 | 20.1 | 5.1 | 250 | 250 | 900 |

**Table S1-b.** Pre-sputtering specifics for SiN_x_

| Step | Target | Gas Ratio | | | Pressure (mTorr) | | RF Power (W) | | Nominal Time (s) |
| --- | --- | --- | --- | --- | --- | --- | --- | --- | --- |
|  |  | Ar (sccm) | N_2_ (sccm) | H_2_ (sccm) | Ignition | Process | Ignition | Process |  |
|  | Ag | 20 | 0 | 0 | 20.1 | 5.1 | 300 | 300 | 60 |
|  | Ag | 8 | 8.6 | 0 | 20.1 | 5.1 | 300 | 300 | 60 |
|  | Si | 100 | 0 | 0 | 25.1 | 5.0 | 50 | 150 | 1800 |
|  | Si | 50 | 50 | 0 | 25.1 | 2.0 | 50 | 200 | 900 |

**Table S2-a.** Deposition specifics for hydrogenated AlN

| Material | Target | Gas Ratio | | | Pressure (mTorr) | | RF Power (W) | |
| --- | --- | --- | --- | --- | --- | --- | --- | --- |
|  |  | Ar (sccm) | N_2_ (sccm) | H_2_ (sccm) | Ignition | Process | Ignition | Process |
| Ag | Ag | 8 | 8.6 | 0 | 20.1 | 5.1 | 300 | 300 |
| AlN | Al | 15 | 3.6 | 0.7 | 20.1 | 5.1 | 250 | 250 |

**Table S2-b.** Deposition specifics for SiNx

| Material | Target | Gas Ratio | | | Pressure (mTorr) | | RF Power (W) | |
| --- | --- | --- | --- | --- | --- | --- | --- | --- |
|  |  | Ar (sccm) | N_2_ (sccm) | H_2_ (sccm) | Ignition | Process | Ignition | Process |
| Ag | Ag | 8 | 8.6 | 0 | 20.1 | 5.1 | 300 | 300 |
| SiN | Si | 50 | 50 | 0 | 25.1 | 2.0 | 50 | 200 |

**UV-Vis**

Perkin Elmer Lambda 1050 UV-Vis-NIR Spectrophotometer was used to carry out the transmittance and reflectance measurements. The transmittance was obtained using the 150mm InGaAs Integrating Sphere Accessory while the film-side reflectance was obtained from the Universal Reflectance Accessory. The optical measurements were carried out at intervals of 5 nm. For measurements between 200nm and 860nm, the response time of the UV-VIS detector is 0.52sec with a gain of 2.5. The baseline measurements of 0% transmittance, 100% transmittance, 0% reflectance, and 100% reflectance were collected before the transmittance and reflectance data. The absorption (A) was inferred from the transmittance (T) and reflectance (R) with the conservation of energy formula: $A=1-T-R.$

**Absorption Spectra**

We observed that both the peak intensity and peak wavelength vary strongly with the thickness of the layers. In samples S2, S6, and S7 (Fig. 1k) – wherein the Ag layer is sandwiched between 20 nm AlN layers on either side – a clear decreasing trend in both peak intensity and peak wavelength is observed with decreasing Ag layer thickness. Further, for the thinnest 18 nm Ag layer (S7), the peak shifts into the UV and appears almost flat, which agrees well with results in the literature for thin 12 nm Ag layers (see Supplementary Information – Diffusion as Loss). For samples S1-S5 with increasing dielectric thickness on bulk silver, the serial modes of constructive and destructive interferences cause the absorption intensities at ~500 nm wavelength to vary cyclically with dielectric thickness (Fig. 1f). These interference maxima and minima remain an important factor in the experimental results (Fig. 1j), wherein the ranking of absorption intensities among samples S1-S5 remain unchanged. However, the observed peaks (Fig. 1j) are much stronger, sharper, and more blue-shifted than those predicted by interference alone (Fig. 1f).

**Scattering matrix method**

This is a well-known rigorous formulation of Maxwell’s Equations for electromagnetic wave propagation in one-dimensional media such as the metal-dielectric multilayer. The method can take in as parameter, a list of refractive indices and their associated thicknesses, and return the optical transmission and reflection coefficients of the multilayer described by the list. Although the method itself is general, in cases such as that of Fig. 1f-i, if the assumption of a simple AlN / Ag / AlN structure and thus the corresponding list of refractive indices and thicknesses is incorrect, the output will not match the experimental results.

**ToF-SIMS**

The sputter time serves as a proxy for film depth only in the sense that the both sputter time and sputter depth increase as the experiment proceeds. It should not be inferred from the data, however, that there is direct correspondence between sputter time and depth (there are many other variables, including material types, ion beam energy, sputter ion identity, current, etc…). We also note that the differences in the ion counts among the various ion species cannot be interpreted as differences in relative concentration; so generally, ion counts could only correspond to the concentration of the species examined. However, proportional conversion between the measured ion counts of a given species and the desired corresponding concentration is only valid for each ion species in regions of similar oxidation states, such as within a material layer. The magnitude of ion counts is contingent on the element and its chemical matrix, so the absolute magnitude of the ion counts at any given sputter time carries no direct information regarding the atomic concentration of the film at that depth level – only the relative magnitude of a particular ion species at different depths may suggest the relative atomic concentration at those depths. Thus, the fits were performed only for the profile of one species at a time. We picked out the appropriate time interval to carry out the fit, with a complimentary error function *f*$(x)$ model defined as:

$$f\left( x \right)=A*erfc[ S\left( x-C \right) ]$$

Wherein the parameters slope S and centre C are defined the same way as in the main text, and the amplitude parameter A is defined for ion count rather than for vol% as in the main text.

The fit results are displayed below:

**Table S3**. Statistics of Fig. 2 *erfc* function fits

| Figure (Sample) | Fit Time Range | Slope, with (initial guess) | Centre, with (initial guess) | Amplitude, with (initial guess) | Goodness of Fit ($\boldsymbol{\chi}^{\mathbf{2}}\boldsymbol{/dof)}$ |
| --- | --- | --- | --- | --- | --- |
| Fig. 2b (S2) | 30-55 | 0.11250 +/- 0.00203 (0.06000) | 42.62159 +/- 0.16580 (36.00000) | 1845.80176 +/- 26.58902 (10000.00000) | 1.59598 |
| Fig. 2c (S2) | 10-85 | 0.05307 +/- 0.00085 (0.06000) | 52.46547 +/- 0.26366 (65.00000) | 1060.09213 +/- 8.86226 (2000.00000) | 2.90594 |
| Fig. 2e (S4) | 10-58 | 0.06704 +/- 0.00140 (0.06000) | 36.01013 +/- 0.31566 (36.00000) | 1578.07440 +/- 24.92965 (2000.00000) | 3.69303 |
| Fig. 2f (S4) | 60-80 | 0.16059 +/- 0.00455 (0.20000) | 68.33332 +/- 0.18268 (68.00000) | 1168.23242 +/- 25.75853 (50000.00000) | 2.10939 |
| Fig. 2h (S5) | 102-114 | 0.24072 +/- 0.00595 (0.06000) | 108.36698 +/- 0.10856 (150.00000) | 5708.37941 +/- 120.48441 (10000.00000) | 5.46518 |
| Fig. 2i (S5) | 103-123 | 0.11633 +/- 0.00405 (0.06000) | 111.08643+/- 0.32843 (65.00000) | 4672.40966 +/- 156.70792 (2000.00000) | 10.78492 |
| Fig. 2k  (S6) | 50-70 | 0.13626 +/- 0.00100 ( 0.06000 | 59.49699 +/- 0.05211 ( 80.00000) | 13292.46966 +/- 72.32722 (20000.00000) | 2.10597 |
| Fig. 2l  (S6) | 30-90 | 0.05793 +/- 0.00114 ( 0.06000) | 63.74432 +/- 0.30850 ( 65.00000) | 4066.15893 +/- 46.14646 (2000.00000) | 19.44231 |

Upon close examination of the fitted results (second column of Fig. 2 of main text), with particular attention to the intervals of sputter time during which *erfc* transitions take place for the plotted silver ion count and plotted aluminum ion count, we find that the two (Ag and Al) intervals are never exactly overlapping; in fact, the intervals are either partially overlapping or disjoint. An exact overlap of the ion count transitions is expected for an ideal interface between two different materials, for which the *erfc* function is always observed due to roughness of the interface (Fig. 1b of main text) and resolution of the detector. In contrast, partially or completely disjoint concentration transitions indicate changes in concentration of one material within the other in addition to the usual transitions at the interfaces; this is most apparent in samples S4 (Fig. 2b of main text). Note that we could have fitted the Ag (or Al) ion counts to two *erfc* functions directly, but the statistics of the fit would be compromised as one of the two *erfc* functions would inevitably suffer from high noise or low signal.

**Calculation of Silver Volume Concentration**

Since the concentration of nitrogen is relatively low (12.6% a.t), we estimate the volume fraction of silver (10.5 g/cm^3^) assuming it to be within an oxygen-rich^[1,2]^ amorphous aluminum oxide (3.77 g/cm^3^)^[3]^. While silver diffusion might be visually apparent in the XPS profile, XPS has a depth resolution of about 10 nm, so the atomic concentrations at a given depth are significantly convolved/blurred. This limits the quantitative analysis of the full diffusion profile of the silver. That said, the measured relative atomic concentration of silver at 1297s (after the vast majority of the carbon contamination is removed) is 0.12%. As can be seen in Fig. 3a, both the top and seed AlN layers are heavily oxidized. In fact, there is significantly more oxygen than nitrogen in the top AlN layer, with the mid-layer Al:O:N ratio being roughly 3:4:1. The oxidation of the AlN has been observed in literature and is attributed to nitrogen hydrolysis^[1,2]^.

**SE of AlN**

**AlN on Si.** Spectroscopic Ellipsometry (SE) was performed on AlN deposited on Si substrate with results in Fig. S1. The complex refractive index (Fig.S1a) has a dispersion relationship characteristic of dielectrics with bandgap in the UV. The fit (Fig.S1b) from which the index was generated show an excellent match between experimental and theory. This complex index of AlN was taken, wherever appropriate, as inputs to theoretical calculations. The thickness of AlN was obtained from the fit and used with sputter time to calculate an approximate rate of deposition for the nominal thicknesses in Table 1 of main text.

**
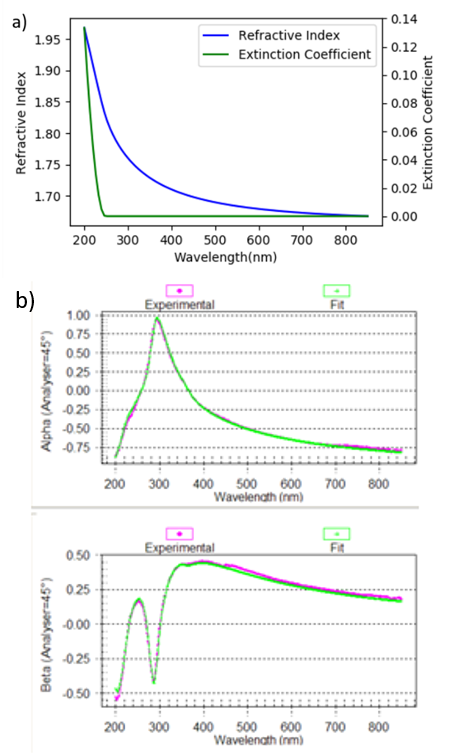
**

**Figure S1 | SE Results 50 nm AlN on Si. (a)** Complex refractive index spectrum from **(b)** the fit of the normalized Fourier coefficients: Alpha and Beta^[4]^.

**Thin AlN on thick Ag.** To obtain the complex refractive indices of the top AlN layer on bulk Ag, Spectroscopic Ellipsometry (SE) was performed on samples S1 and S2. We chose only these two samples because:

1. The silver, at 300 nm, is optically thick.
2. There is little upward diffusion of Ag in these samples (as was shown in the ToF-SIMS profile of S2).
3. The top layer thickness is of the order of the interface roughness (S1: 3 nm, S2: 20 nm, roughness: ~15 nm), which allows for representation of the top layer as one layer with a homogenous effective refractive index spectrum.

Using the ellipsometry analysis software Winelli II, a model consisting of 4 Lorentz Oscillators^[4]^ was used to successfully fit the ellipsometric data. Fig. S2,3 and Table S4,5 below show the regression fit results of ellipsometry on samples S1 and S2 of Table 1, respectively. The Lorentz Oscillator is a commonly used dielectric function that models the electron-nucleus interaction under incident electromagnetic waves as a forced ball-spring oscillator. It computes a dielectric constant dispersion $\epsilon(\lambda)=\epsilon_{r}(\lambda)-j\epsilon_{i}(\lambda)$ of^[4]^:

$$\epsilon_{r}\left( \lambda\right)=\frac{A\lambda^{2}(\lambda^{2}-\left( \frac{K_{0}}{E_{0}} \right)^{2})}{\left( \lambda^{2}-\left( \frac{K_{0}}{E_{0}} \right)^{2} \right)^{2}+\Gamma^{2}\lambda^{2}}$$

$$\epsilon_{i}(\lambda)=\frac{A\lambda^{3}\Gamma}{\left( \lambda^{2}-\left( \frac{K_{0}}{E_{0}} \right)^{2} \right)^{2}+\Gamma^{2}\lambda^{2}}$$

For the constant $K_{0}=1.24 eV$and the three parameters A, $E_{0}$ and $\Gamma$ which represent the intensity, central energy, and width of the peak, respectively. We choose to set the locations of these oscillators near peaks and dips of the UV-Vis absorption spectra (see Fig. 1 of main text) to model those behaviors. Note in Table S4,5 that one of the Lorentz oscillators in the four Lorentz oscillator model has negative intensity which corresponds to a dip instead of a peak. This was a trick to fit the sharp absorption dip in the transition region between UV and visible.

| 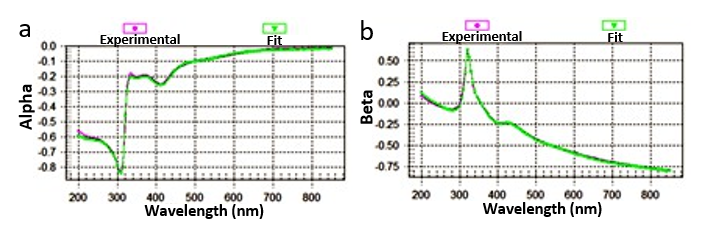 |
| --- |
| **Figure S2.** Regression fit to the experimental ellipsometry data of 3 nm AlN / 300 nm Ag / 20 nm AlN / Si (Sample S1). (a) Alpha, and (b) Beta are normalized Fourier coefficients^[4]^. Fit results are summarized in Table S4.  **Table S4.** Best fit parameters used in dispersion model using 4 Lorentz Peaks for fitting 3 nm AlN / 300 nm Ag / 20 nm AlN / Si sample (Sample S1 of Table 1).   \| Peak Number \| Model \| Intensity A \| Energy of Central Wavelength E_0_ (μm) \| Width of Peak (μm) \| \| --- \| --- \| --- \| --- \| --- \| \| 1 \| Lorentz \| -0.300± 0.127 \| 0.330 ± 0.005 \| 0.0741 ± 0.0220 \| \| 2 \| Lorentz \| 3.54 ± 0.436 \| -0.00221 ± 1.20570 \| 0.138 ± 0.018 \| \| 3 \| Lorentz \| 1.32 ± 0.14 \| 0.412 ± 0.002 \| 0.0871 ± 0.0067 \| \| 4 \| Lorentz \| 0.314 ± 0.099 \| 0.545 ± 0.010 \| 0.112 ± 0.035 \|   The Thickness (μm) parameter used in the model was: 0.00564 ± 0.00026  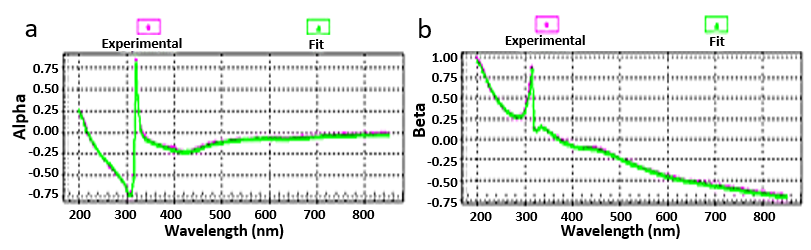 |

**Figure S3.** Regression fit to the experimental ellipsometry data of 20 nm AlN / 300 nm Ag / 20 nm AlN / Si (Sample S2). (a) Alpha, and (b) Beta are the normalized Fourier coefficients^[4]^. Fit results are summarized in Table S5.

**Table S5.** Best fit energy parameters used in dispersion model using 4 Lorentz Peaks to fit 20nm AlN / 300 nm Ag / 20 nm AlN / Si sample (Sample S2 of Table 1).

| Peak Number | Model | Intensity A | Energy of Central Wavelength E_0_ (μm) | Width of Peak$\boldsymbol{\Gamma}$ (μm) |
| --- | --- | --- | --- | --- |
| 1 | Lorentz | -0.0785± 0.0119 | 0.339 ± 0.002 | 0.0392 ± 0.004 |
| 2 | Lorentz | 2.70 ± 0.06 | 0.0736 ± 0.0035 | 0.0354 ± 0.0009 |
| 3 | Lorentz | 0.544 ± 0.037 | 0.436 ± 0.002 | 0.114 ± 0.008 |
| 4 | Lorentz | 0.107 ± 0.031 | 0.654 ± 0.012 | 0.126 ± 0.042 |

The Thickness (μm) parameter used in the model was: 0.0170 ± 0.0003

The resulting complex refractive index spectra, as computed from the best fit of the 4 Lorentz Oscillator model, are shown in Fig. S4a,b. They do not resemble that of the pure AlN dielectric (Fig. S1a) nor that of a simple roughness layer made up of an equal mixture of AlN and Ag. Instead, they exhibit the anomalous dispersion characteristic of plasmonic materials^[5,6]^.

Using the refractive index of the modelled layer, we can compute the absorption spectra of S1 and S2 as shown in Fig. S4c,d using the scattering matrix method. The resulting spectra agree very well with the experimental spectra.


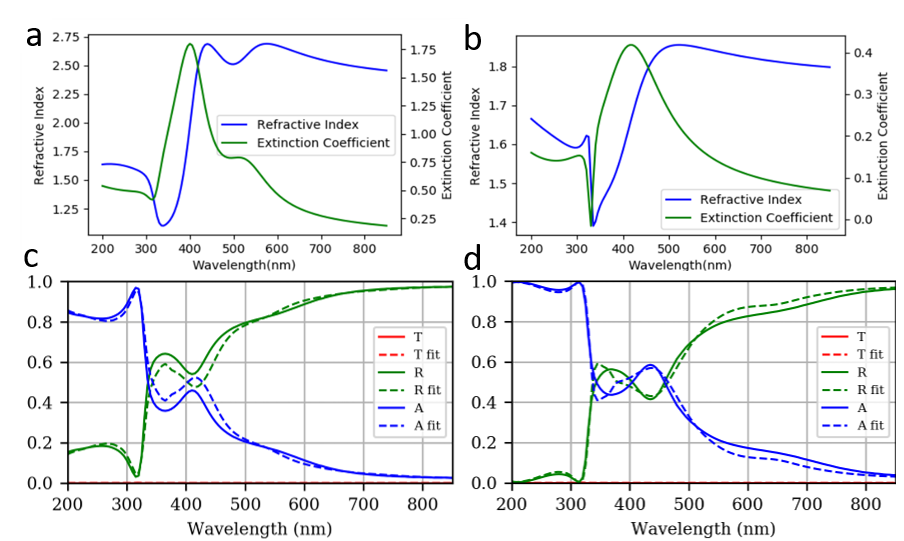


**Figure S4 | Effective index and calculated absorption spectra for S1 and S2.** The refractive indices and the extinction coefficient of **(a)** Top layer of Sample S1 (3 nm AlN / thick Ag) and **(b)** Top layer of Sample S2 (20 nm AlN / thick Ag) obtained with a 4-Lorentz fit to the SE data. The calculated absorption spectra of **(c)** S1 and **(d)** S2 using the indices from SE fit and the Scattering matrix method.

**Roughness LSPR**

It is noted that LSPR can indeed be excited on the tips of roughness features (the top of the ‘hills’ of the surface terrain) which have high curvature and are in relative isolation. After all, the hills and valleys of a film surface are commonly modelled as a conglomerate of nanoparticles. Thus, a natural challenge to the proposed MG / BG hybrid model may be the apparent lack of consideration for this roughness induced LSPR effect. However, the proposed model does account for this effect by lumping it together with the diffusion induced LSPR region. As the division line between the MG and the BG modelled regimes (for the AlN samples) is determined from the regression fit, the best fit results would ideally draw the line such that the highly curved hills of the roughness features are lumped with the diffused nanoparticles just above them, so that all relevant LSPR sources are accounted for. For the SiN_x_ samples, where there is no regression fit and the selection of the LSPR vs SPR division line is solely based on experimental concentration, we can be reasonably confident that we’ve captured all the LSPR by considering all regions with relatively low (taken to be 0.1 vol%) Ag concentration.

Furthermore, one can entertain the hypothesis that the nanoparticle diffusion originate from the detachment of roughness features due to surface energy minimization, and thus the tip of the rough silver surface serves as the reservoir of nanoparticles. In this model, the lumping of the diffused nanoparticles with the hills of the silver surface is not only a convenient mathematical formulation, but also captures the physical picture that the LSPR-inducing roughness features are just nanoparticles at time zero of the diffusion process.

**Samples S1 and S2**

Note that we do not apply our model to samples S1 and S2 because these samples have dielectric thickness of less than 20 nm, which is of the same order as the thickness of the roughness features of the 300 nm silver layer. With extremely limited distances for silver nanoparticles to diffuse, the strong coupling (see Supplementary Information -- Absorption Fit from Diffusion) between the diffused nanoparticles and the nearby roughness features would make the model’s separation of the MG and BG regimes break down. For these samples, we instead treat the dielectric and its embedded silver as one homogenous layer and perform spectroscopic ellipsometry for the refractive index. The fit and results can be found in Supplementary Information – SE of AlN. The absorption spectra calculated from the best fit refractive index, match very well with the experimental spectra. However, we stress that the SE fits are strictly mathematical as they do not model the complex physical setup involving diffusion and roughness, and the single homogenous layer assumption does not hold for thicker dielectric layers in which the gradient of silver content must be accounted for. Additional diffusion effects beyond the top dielectric layer were not considered. This means that we did not alter the thickness of the silver layer to account for the losses due to diffusion, and we kept the second interface of Ag / AlN toward the substrate as an ideal sharp interface. These assumptions are very well justified for thick silver layer samples, as the thick silver is optically thick and functions effectively as the substrate. Practically, this keeps the model simple and the regression fit clean with less ambiguous results. But we can see from the ToF-SIMS and XPS results in the main text that these assumptions are not as well-justified for the thin silver sample, S6. Consequently, the errors on the best-fit parameters of S6 (see Table S5) are much greater compared to the other samples. An important area of future work would be to specialize the model for multilayers comprised of thinner silver layers, and/or multilayers with multiple silver layers, wherein the diffusion at every interface as well as the individual silver layer thicknesses must be added to the model as parameters.

**Absorption Fit from Diffusion**

The fill factor of silver in the top dielectric layer, $f\left( x \right)$, was constructed as in Eqn. 1 of main text. In the regression fit algorithm, six parameters were used fully constrain $f\left( x \right)$, as shown in Table S6. The first is “Erf_scale” which scales the relative amplitude A_1_ and A_2_ of the first and the second $erfc\left( x \right)$. The first, smaller amplitude $erfc_{1}\left( x \right)$ will have some A_1_, and the second, larger amplitude $erfc_{2}\left( x \right)$ will have A_2_ = 0.5 – A_1_ in order to satisfy the 0% and 100% end behaviours of $f\left( x \right)$. Then, “S_1_”, “C_1_”, “S_2_” are defined as they appear in Eqn. 1. The parameter “C_2_ – C_1_” is confined to be positive (to avoid two identical solutions under the exchange C_1_ <-> C_2_) and defines C_2_ relative to C_1_. Finally, the parameter L is the depolarization coefficient as it appears in the Maxwell Garnett Effective Medium Theory formula.

The MG model assumes spherical inclusions of a metal with fractional content $f$and dielectric constant $\varepsilon_{M}$ within a dielectric host with fractional content $1-f$and dielectric constant $\varepsilon_{D}$, such that $f$ is small and below 30%. The depolarization coefficient *L* affects the peak absorption wavelength significantly, and it is because of the difference in the best fit depolarization coefficients (see Table S6) that the resulting peak wavelength at the same Ag concentration varies among the different samples in Fig. 4 of the main text.

We note that thin film effects can play a significant role in the absorption spectra, which are not visually highlighted in the Fig 4i-l contour plots (in the main text) but are accounted for in the scattering matrix method calculations. This is the most apparent when contrasting the “absorption triangles” of samples S3 and S4. We might expect similar absorption spectra with S3 having higher absorption coefficient values but S4 having more absorption distance (note that the distance axis is re-scaled for different samples), but the actual absorption spectra of S3 has significantly higher peak absorption intensity (at ~80%) compared to S4’s (at ~60%). This is because S3 and S4 exhibit interference maxima and minima, respectively.

An important caveat in Fig. 4 (of the main text) is that the slope of the first *erfc* function closest to the expected silver surface may appear too steep to accurately represent the ~15 nm of silver roughness of the thick Ag samples (which is the total thickness of the roughness layer, not the root mean square roughmess. See the Roughness Estimation section of Supplementary Information for details). This is because the BG modelled first *erfc* function region cannot excite LSPR, and thus has very limited contributions to the absorption spectra. This means that this region is too optically similar to the underlying bulk silver for the regression algorithm to distinguish, which is reflected in the fit statistics as the high uncertainty in this slope parameter (see Table S6).

**Table S6.** Best fit results of Samples S3-6 corresponding to Fig. 4 of main text.

| Fit Parameters | A_1_, with (initial guess) | S_1_, with (initial guess) | C_1_, with (initial guess) | S_2_, with (initial guess) | C_2_ – C_1_, with (initial guess) | L, with (initial guess) |
| --- | --- | --- | --- | --- | --- | --- |
| S3 (50 nm AlN / thick Ag) | 0.13876 +/- 0.01639 (0.12000) | 0.21723 +/- 0.07035 (0.33000) | 25.87734 +/- 1.08659 (23.60000) | 0.54908 +/- 0.23744 (0.10000) | 9.18657 +/- 0.57226 (12.50000) | 0.54052 +/- 0.01922 (0.53000) |
| S4 (100 nm AlN / thick Ag) | 0.05807 +/- 0.03958 (0.05000) | 0.26445 +/- 0.00001 (0.15000) | 51.84749 +/- 2.83977 (40.00000) | 0.49478 +/- 0.55569 (0.80000) | 7.43827 +/- 3.06790 (14.00000) | 0.41670 +/- 0.01196 (0.35000) |
| S5 (200 nm AlN / thick Ag) | 0.00836 +/- 0.00000 (0.00018) | 0.35783 +/- 0.10447 (0.06052) | 48.36705 +/- 5.49889 (47.76848) | 0.81224 +/- 0.30724 (0.76512) | 15.39151 +/- 5.27363 (16.88951) | 0.35826 +/- 0.00495 (0.38077) |
| S6 (20 nm AlN / 25 nm Ag / 20 nm AlN) | 0.08637 +/- 0.07447 (0.15000) | 0.17317 +/- 0.07703 (0.16000) | 51.27958 +/- 9.67943 (70.00000) | 1.54391 +/- 19.51977 (0.60000) | 7.11913 +/- 12.46078 (5.00000) | 0.54375 +/- 0.05104 (0.60000) |

The model of two *erfc* functions with MG and BG EMT succinctly captures the absorption features in our samples and is physically realistic. However, owing to the assumptions inherent to these two EMTs^[7]^, it may omit some second order effects such as additional enhanced coupling that results from adjacent nanoparticles as well as between the nanoparticles and the roughness features of the metal surface. The lack of a fair treatment of the latter is a well-known problem^[8]^ from the discontinuity between the MG and BG EMT formalisms^[9]^, and manifests as the “sheath” or the sudden transition between the SPR and LSPR dominant regions in the contour plots of main text Fig. 4. Nevertheless, the success of the model in producing the observed absorption spectra is convincing evidence that the diffusion process and the consequent LSPR phenomenon associated with Ag nanoparticles within the dielectric is crucial for the observed coloration of the films. The model succeeds in predicting the absorption peaks which previously could not be explained by interference and roughness effects alone (as shown in main text Fig. 1f-i).

**Roughness** **Estimation**

We estimate the thickness of the rough silver-AlN interface by examining various TEM images (Fig. S5) and determining the distribution of peak to valley heights (Fig. S6). The height, averaged over the sum of roughness features seen in Fig. S5, is 14.61 nm which is rounded up to 15 nm for EMT calculations in Fig. 1 of main text.

Note that this is an estimate of the peak-to-valley height of the roughness features, and not the root mean squared roughness values obtained from Atomic Force Microscopy (AFM), which are often quoted in the literature. This value is more convenient and sufficient for us because we model the entire rough region as a single mixed layer.


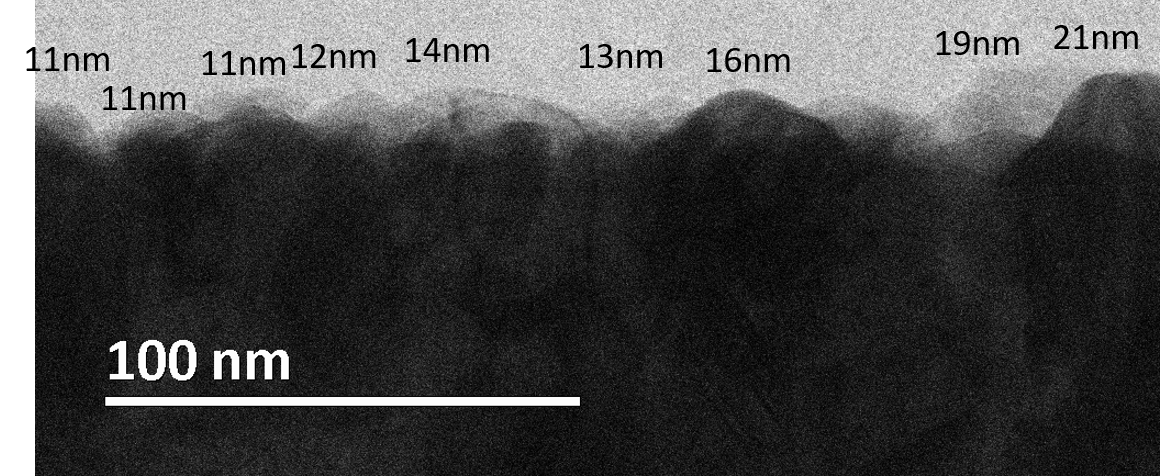


**Figure S5.** A cross-sectional TEM image of the top AlN/Ag interface in sample S5. The heights of the various features are denoted.


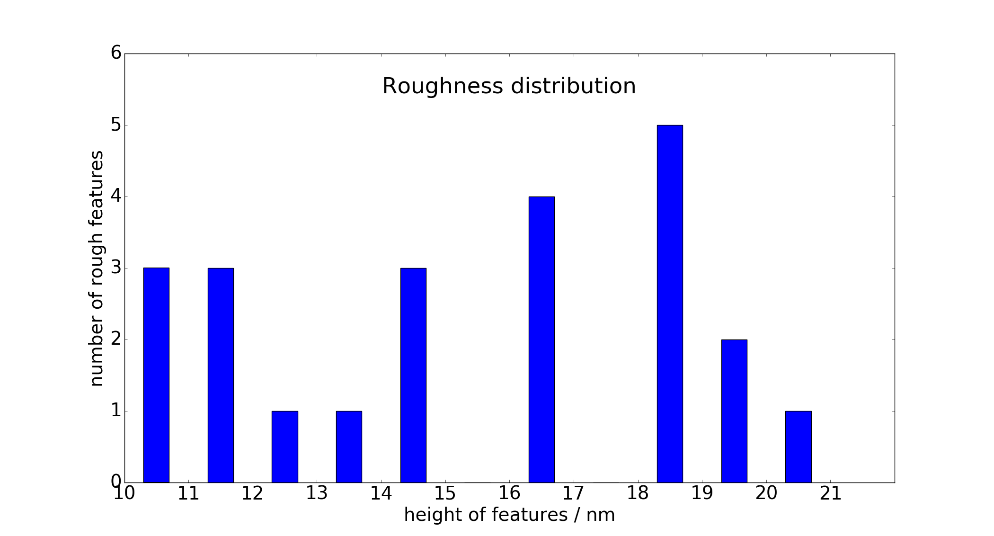


**Figure S6**. The distribution of roughness features.

**SE of SiN_x_**

To determine the thickness and refractive index of our SiN_x_ films, Spectroscopic Ellipsometry (SE) was performed. Silicon substrates were used, the deposition parameters were adjusted until we had a film close to 200nm thick. The data was fitted to a Tauc-Lorentz model (R^2^=0.994) and the resulting parameters for the 200nm samples are shown in Table S7. The resulting complex refractive index (Fig. S7) was taken, wherever appropriate, as inputs to theoretical calculations.

**Table S7.** Best fit results of a SiN film on silicon substrate using the same deposition parameters as those for the SiN / Ag / SiN sample analyzed in the main text.

| Parameter | Value |
| --- | --- |
| Thickness (µm) | 0.186 ± 0.001 |
| ε_r_(∞) | 2.26 ± 0.09 |
| E_g_ (eV) | 3.71 ± 0.12 |
| E_0_ (eV) | 7.65 ± 0.13 |
| C (eV) | 1.31 ± 0.17 |
| A | 42.0 ± 4.6 |

**Figure S7 | Effective index and calculated absorption spectra for SiN_x_**. This graph corresponds to the fit values given in Table S7.

**Diffusion as Loss**

For our AlN / Ag / AlN system (with a thin 20 nm AlN top layer), the visible absorption peak is prominent for silver thickness greater than a moderate 18 nm, and the UV absorption tail is present for all silver thicknesses tested (Fig. 1k). These metal thicknesses are relevant in transmission coatings designed for visible^[10]^ and UV^[11]^ ranges, so designers may be informed to consider diffusion mitigation methods such as alloying^[12,13],[14]^.

**References**

[1] X. D. Wang, K. W. Hipps, U. Mazur, *Langmuir* **1992**, *8*, 1347.

[2] Y.-J. Yong, J.-Y. Lee, *J. Vac. Sci. Technol. A Vacuum, Surfaces, Film.* **1997**, *15*, 390.

[3] P. Juliet, K. Koski, J. Holsa, *Thin Solid Films* **1999**, *339*, 240.

[4] H. Fujiwara, *Spectroscopic Ellipsometry Principles and Applications*, **2007**.

[5] D. Magnfält, E. Melander, R. D. Boyd, V. Kapaklis, K. Sarakinos, *J. Appl. Phys.* **2017**, *121*, DOI: 10.1063/1.4979139.

[6] B. Gao, M. J. Rozin, A. R. Tao, *Nanoscale* **2013**, *5*, 5677.

[7] G. A. Niklasson, C. G. Granqvist, O. Hunderi, *Appl. Opt.* **1981**, *20*, 26.

[8] J. Szcyrbrowski, K. Schmalzbauer, H. Hoffmann, *Thin Solid Films* **1985**, *130*, 57.

[9] J. I. Gittleman, B. Abeles, *Phys. Rev. B* **1977**, *15*, 3273.

[10] B. von Blanckenhagen, D. Tonova, *Proc. SPIE* **2005**, *5963*, 596317.

[11] Z. Wang, Q. Chen, X. Cai, *Appl. Surf. Sci.* **2005**, *239*, 262.

[12] K. Chiba, S. Kaminishi, *Jpn. J. Appl. Phys.* **2008**, *47*, 240.

[13] K. Chiba, K. Suzuki, *Sol. Energy Mater. Sol. Cells* **1992**, *25*, 113.

[14] M. E. A. Warwick, R. Binions, *J. Mater. Chem. A* **2014**, *2*, 3275.
